# Beneficial Role of *Carica papaya* Extracts and Phytochemicals on Oxidative Stress and Related Diseases: A Mini Review

**DOI:** 10.3390/biology10040287

**Published:** 2021-04-01

**Authors:** Yew Rong Kong, Yong Xin Jong, Manisha Balakrishnan, Zhui Ken Bok, Janice Kwan Kah Weng, Kai Ching Tay, Bey Hing Goh, Yong Sze Ong, Kok Gan Chan, Learn Han Lee, Kooi Yeong Khaw

**Affiliations:** 1 Biofunctional Molecule Exploratory Research Group (BMEX), School of Pharmacy, Monash University Malaysia, Bandar Sunway 47500, Malaysia; ykon0007@student.monash.edu (Y.R.K.); yjon0001@student.monash.edu (Y.X.J.); mbal0001@student.monash.edu (M.B.); zbok0001@student.monash.edu (Z.K.B.); janicekwan97@gmail.com (J.K.K.W.); kctayyy@gmail.com (K.C.T.); goh.bey.hing@monash.edu (B.H.G.); Ong.YongSze@monash.edu (Y.S.O.); 2 College of Pharmaceutical Sciences, Zhejiang University, Hangzhou 310058, China; 3 Division of Genetics and Molecular Biology, Faculty of Science, Institute of Biological Sciences, University of Malaya, Kuala Lumpur 50603, Malaysia; 4 Institute of Marine Sciences, Shantou University, Shantou 515063, China; 5 Novel Bacteria and Drug Discovery Research Group (NBDD), Microbiome and Bioresource Research Strength (MBRS), Jeffrey Cheah School of Medicine and Health Sciences, Monash University Malaysia, Bandar Sunway 47500, Malaysia

**Keywords:** oxidative stress, antioxidant, *Carica papaya*, inflammation, diabetes, cancer, aging, wound healing, periodontal disease, Alzheimer’s disease

## Abstract

**Simple Summary:**

This review highlights the medicinal benefits of a natural remedy, the *Carica papaya* extracts and its phytochemicals. In this review, the potential of *Carica papaya* against various conditions, including cancer, inflammation, aging, healing of the skin, and lifelong diseases has been summarized and discussed. In short, more research and development should focus on this natural remedy that can potentially act as a prophylaxis against chronic diseases.

**Abstract:**

Oxidative stress is a result of disruption in the balance between antioxidants and pro-oxidants in which subsequently impacting on redox signaling, causing cell and tissue damages. It leads to a range of medical conditions including inflammation, skin aging, impaired wound healing, chronic diseases and cancers but these conditions can be managed properly with the aid of antioxidants. This review features various studies to provide an overview on how *Carica papaya* help counteract oxidative stress via various mechanisms of action closely related to its antioxidant properties and eventually improving the management of various oxidative stress-related health conditions. *Carica papaya* is a topical plant species discovered to contain high amounts of natural antioxidants that can usually be found in their leaves, fruits and seeds. It contains various chemical compounds demonstrate significant antioxidant properties including caffeic acid, myricetin, rutin, quercetin, α-tocopherol, papain, benzyl isothiocyanate (BiTC), and kaempferol. Therefore, it can counteract pro-oxidants via a number of signaling pathways that either promote the expression of antioxidant enzymes or reduce ROS production. These signaling pathways activate the antioxidant defense mechanisms that protect the body against both intrinsic and extrinsic oxidative stress. To conclude, *Carica papaya* can be incorporated into medications or supplements to help manage the health conditions driven by oxidative stress and further studies are needed to investigate the potential of its chemical components to manage various chronic diseases.

## 1. Introduction

Oxidative stress is a natural phenomenon, resulting from the disruption of the redox equilibrium due to the amount of pro-oxidants outweighing antioxidants, which can eventually result in cell or tissue damage. As the name suggests, either oxidative stress can be induced by the presence of a high amount of pro-oxidants or the incompetence of antioxidant defense mechanism in the human body. In normal circumstances, human body is capable of scavenging free radicals, inhibiting the generation of oxidative stress with the help of several antioxidant enzymes, including glutathione peroxidase (GPx), superoxide dismutase (SOD), and catalase (CAT). Among various pro-oxidants, those free radicals that contain oxygen are known as reactive oxygen species (ROS), and ROS are considered as the secondary products of aerobic metabolism. Examples of ROS include singlet oxygen, hydroxyl radicals, superoxide radicals, and hydrogen peroxide. Sources of free radicals include diet, environment, and sunlight exposure and their accumulation can lead to oxidative stress and tissue injury, consequently leading to aging of the skin and medical conditions, including Alzheimer’s disease (AD), rheumatoid arthritis, asthma, atherosclerosis, and cancers [1,2]. Various researches have been conducted to investigate the pathophysiology of diseases related to oxidative stress and the benefits of antioxidants in treating those diseases. Antioxidants can be found abundantly in plants. An example of antioxidant-rich plant is the *Carica papaya* L., which is a flowering and dicotyledonous plant, classified as violales order, *Caricaceae* family, *Carica* L. genus, and papaya species [3]. The *Carica papaya* L. has a single hollow light greenish to brownish stem with scarring, bearing big leaves and big oval fruits. Besides, this plant is cultivated in countries, such as Malaysia, Brazil, South America, Australia, and Indonesia, which are located near to the equator. The *Carica papaya* L. plant is known as many different names, such as kepaya, paw paw, or tapaya, based on its geographical distribution. In fact, this plant is acclaimed for an array of medicinal values from each part of the plant including fruit, roots, leaves, and seeds of the plant. Therefore it has been used as a traditional treatment regimen for various diseases [4]. Some of the medicinal properties of the plant can be explained by its antioxidative property, which confer protection on the cells from being harmed by oxidative stress [5]. Papain is the most widely exploited proteolytic enzyme from the *Carica papaya* L. and it has been used to help with meat tenderization and digestion. It is worth to note that papain exhibited great potential as a medication [4], as it is suggested to exhibit drug-like properties for atherosclerosis and associated conditions, which involve monocyte-platelet aggregate (MPA)-regulated inflammation [6]. Relevant and significant studies have been conducted to evaluate the benefits of the *Carica papaya* extracts and chemical constituents. This review aims to gather and summarize the research findings linking the *Carica papaya* to its antioxidant properties and the utilization of this natural resource as a pharmaceutical, cosmeceutical, and nutraceutical products.

## 2. Methods

All literature was retrieved from databases (PubMed, Semantic Scholar, Web of Science, WorldWideScience, and Embase) using search terms, including “*Carica papaya*”, “inflammation”, “cancer”, “Alzheimer’s Disease”, “diabetes”, “aging”, “wound healing” and “oxidative stress”. Literatures published from 2000 to 2020, investigating the benefits of the *Carica papaya* plant towards various conditions, were included. Literatures that were not related to oxidative stress mechanisms were excluded. Literatures selected were categorized based on related conditions including inflammation, cancer, skin aging, wound healing, diabetes, periodontal diseases, and Alzheimer’s disease (AD). The mechanisms of action of the *Carica papaya* towards each condition were also presented in tables in the respective sections.

## 3. *Carica papaya* Counteracts Oxidative Stress in Inflammation, Skin Aging, and Healing, Chronic Diseases, and Cancers

**Figure 1:**
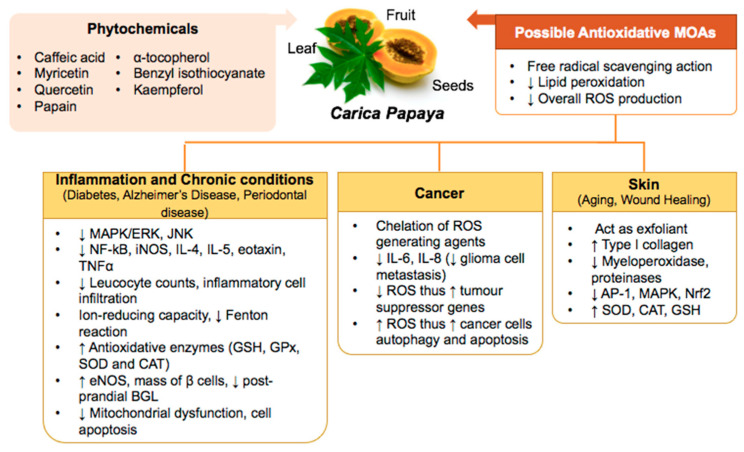
Role of oxidative stress in different medical conditions.

Oxidative stress occurs due to excessive ROS production, which will cause oxidative damage to tissues. Consequence effects of oxidative stress has known to cause inflammation, leading to the development of various health conditions, including AD, rheumatoid disease, cardiovascular diseases (CVDs), cancers, cataracts, as well as cosmetic issues, such as the formation of wrinkles and loss of elasticity of the skin [7,8]. Figure 1 provides an overview of the role of oxidative stress in these conditions.

### 3.1. Inflammation

Inflammation is a complicated pathway of the body’s own protective mechanism against pathogens, which is associated with symptoms such as pain, swelling, and redness due to the release of a mediator “prostaglandin” [9]. This defensive action can be divided into innate and adaptive responses [10]. In short, the pathogenesis of inflammation starts with tissue injury, which causes infiltration and activation of macrophages and relevant antigen-presenting cells (APCs). This causes the release of proinflammatory cytokines such as tumour necrosis factor-α (TNF-α) and interleukins (ILs). Cytokines stimulate the release of chemokines, which further recruit and activate lymphocytes and leukocytes. ROS are produced to eliminate invaders whereby activates Nuclear factor kappa-B (NF-*κ*B). NF-*κ*B is a transcription factor and plays a role in inducing inducible nitric oxide synthase (iNOS) activity and, thus, nitric oxide (NO) production. Excessive ROS upregulated prostaglandin E2 (PGE2) synthesis and, hence, cyclooxygenase-2 (COX-2) expression, which eventually leads to oxidative stress that causes tissue damage and worsens inflammation [11,12,13].

Another study further suggested that oxidative stress and inflammation are interrelated as oxidative stress resulting from high ROS can precipitate the formation of inflammation by increasing the gene expression coding for inflammatory proteins, including NF-*κ*B, peroxisome proliferator activator receptor gamma (PPAR-γ), and activator protein 1 (AP-1). Consequently, inflammatory chemokines and cytokines are produced to induce inflammation. On the other hand, inflammation can increase ROS production via several signaling cascades. Polymorphonuclear neutrophils (PMN) is an immune cell that is largely involved in inflammatory processes. During inflammation, they congregate the gp91-phox, which is a catalytic subunit of NADPH oxidase 2 (NOX) and generate more ROS, including hydroxyl radical, superoxide anion, and hypochlorous acid, thereby enhance inflammation through mitogen-activated protein kinase (MAPK), protein kinase C (PKC), and c-Jun-N-terminal kinase (JNK) pathways [14]. Activation of these signaling cascades lead to production of more inflammatory chemokines and cytokines. Therefore, this forms a vicious cycle leading to chronic inflammation and eventually a range of medical conditions, including cardiovascular diseases, neurodegenerative diseases, and cancers [12,14].

Table 1 shows anti-inflammatory activities of *Carica papaya*. Different parts of *Carica papaya* possess anti-inflammatory effects. Aqueous extract of *Carica papaya* seeds significantly reduced NO radical by 69.4% in a cell free assay in vitro. Meanwhile, the aqueous extract at a concentration of 150 μg/mL inhibited the release of lysosomal enzymes and stabilized human red blood cell membrane by 22.7%. On the contrary, the extract exhibited least potent hydroxyl radical scavenging action (69.1%) at a concentration 95 mg/mL and reducing power at a concentration of 20 mg/mL [15]. Meanwhile, Aruoma and colleagues demonstrated that fermented papaya preparation (FPP) inhibited H_2_O_2_-induced phosphorylation of Akt and p38, as well as downregulating MAPK pathway [16]. An in vivo study showed that *Carica papaya* leaf extract at a dose of 1.32 μg/mL demonstrated immune modulation properties [17].

Somanah and co-workers revealed that papaya extracts at a dose of 2 mg/mL showed protective effects through attenuated ROS production and pro-inflammatory cytokines secretion of interleukin-6 (IL-6) and TNF-α as well as upregulating antioxidant enzymes activities [18]. Another in vivo study showed that papaya juice demonstrated anti-obesity properties by reducing obesity markers, inflammation and oxidative stress in high-fat diet rats by upregulating SOD levels, attenuated serum malondialdehyde (MDA), PPAR-γ, lipid peroxidation, and ROS production at a treatment dose of 1 mL per 100 g of body weight [19].

The anti-inflammatory effect of *Carica papaya* was further investigated on various in vivo experimental studies. For instance, ethanolic extract of *Carica papaya* leaves was found to reduce paw edema induced by carrageenan, granuloma formation, as well as inflammation in formaldehyde-induced arthritis rats at doses of 25–200 mg/kg [20]. Methanolic extract of *Carica papaya* seeds at dosage range of 50 to 200 mg/kg exhibited anti-inflammation activities in egg albumin induced inflammation on Wistar albino rats [21]. Similarly, the aqueous extract of *Carica papaya* seeds at a dose of 400 mg/kg showed anti-inflammatory in carrageenan and formalin induced pedal edema rats [22].

High phenolic and flavonoid content in papaya seed extracts were proposed to act as free radical scavengers and metal ion chelators [15]. Phytochemicals including tocopherols and quercetin are showed to enhance AMP-activated protein kinase (AMPK) activation, as well as the inhibition of COX-2 expression [17]. In addition, a range of phytochemicals with great strength of anti-inflammatory effect, such as benzyl isothiocyanate (BiTC), β-carotene, lycopene, and vitamin C could be found in various parts of papaya fruits, in either pulp or seeds. These phytochemicals were proven to inhibit pro-inflammatory cytokines including TNF-α, IL-6 and monocyte chemoattractant protein-1 (MCP-1) [18,19]. In addition, polyphenols within *Carica papaya* could act as free radical scavenger and at the same time exerting its effects in upregulating the antioxidant enzymes activities [18].

### 3.2. Diabetes

Diabetes is a chronic disease, predominantly due to the insulin resistance or insulin insufficiency phenomenon, which leads to elevation of blood glucose level, a condition known as hyperglycemia [23]. Uncontrolled diabetes can lead to various macro and microvascular complications in which ultimately affect the quality of life of diabetes patients. It has been shown that oxidative stress plays an important role in diabetes and its progression [24]. There are tremendous amount of evidences revealed that uncontrolled hyperglycemia might induce oxidative stress by promoting ROS production and weakening antioxidant defenses via several mechanisms, including inducing lipid peroxidation of low-density lipoprotein (LDL), glycation of proteins, and glucose oxidation. Non-enzymatic interaction of glucose with proteins generates advanced glycation end products (AGEs), and increases nitric oxide (NO). Excessive free radicals can cause dysfunction of β-cells of the islets of Langerhans of pancreas and lead to complications. Thus, these findings support the role of antioxidants in diabetic control [25,26]. Table 2 shows anti-diabetic activities of *Carica papaya*. Agada and co-workers demonstrated that the ethyl acetate extract of *Carica papaya* seeds significantly reduced postprandial glucose levels in streptozotocin-induced diabetic rats. Along with in vivo study, ethyl acetate showed α-glucosidase and α-amylase enzyme inhibitory effect and antioxidant activities in vitro, whereas hexane extract exhibited slightly more potent enzyme inhibitory activities [27].

*Carica papaya* FPP extract showed protective effect against diabetic complications such as atherosclerotic plaque formation, upregulated SOD level and ameliorated lipid peroxidation at a concentration of 50 μg/mL. In addition, increasing platelet membrane fluidity of diabetic patients and preventing chronic hyperglycemia-induced platelet malfunction [28]. Likewise, Somanah and colleagues conducted a study on the impact of short-term supplementation of fermented papaya preparation (FPP) on the biomarkers of diabetes mellitus. The randomized controlled trial showed that daily consumption of 6 g of FPP for a period of 14 weeks enhanced the antioxidant status of the subjects and improved general health status of several organs that were potentially at risk of damage from diabetes. In addition, the FPP extract reduced the aspartate transaminase (AST) and alanine aminotransferase (ALT) levels to enhance insulin sensitivity of the liver and stabilize blood glucose level in diabetic patients [29]. Furthermore, a continuous human trial conducted by Somanah and co-workers showed that the supplementation of FPP successfully reduced erythrocyte hemolysis rate in pre-diabetics. FPP possessed hydroxyl-quenching properties that could possibly prevent DNA damage and boost the total phenolic content that exhibited antioxidant activities [30].

Unripe papaya has been used as a folk medicine, e.g., to relieve menstrual pain, improve ingestion, wound healing, and heart disease. An in vitro study showed that unripe *Carica papaya* fruit inhibited α-amylase and α-glucosidase enzymes. In addition, the fruit extract protected β-cell against oxidative stress in streptozotocin induced diabetes rats. The phytochemical analysis of *Carica papaya* fruits revealed the presence of phytochemicals, including kaempferol, quercetin, and caffeic acid [31].

An in vivo study suggested that chloroform extract of *Carica papaya* leaves protected β-cells of islet of Langerhans from oxidative stress-induced damage and promoted pancreatic β-cells regeneration at a dose of 31 mg/kg, leading to an increase in insulin production [32]. In addition, Juarez-Rojop and colleagues reported that *Carica papaya* leaf extract stimulated the healthy β-cells to release more insulin in vivo. At concentrations of 0.75 and 1.5 g/100 mL, *Carica papaya* leaf aqueous extract also demonstrated antioxidant properties via increasing NO production, consequently lowering ROS production, and diminishing diabetes-induced oxidative stress. As a result, this mechanism delayed or prevented the progression to diabetic complications, such as neuropathy and nephropathy [33].

### 3.3. Alzheimer’s Disease (AD)

Oxidative stress is correlated with the induction and progression of Alzheimer’s disease (AD). AD is manifested by generation of neurofibrillary tangles and an aggregation of β-amyloid peptides in the brain [34]. As the amount of β-amyloid accumulates, it generates ROS that causes lipid and protein peroxidation in the brain, and resultant in neurotoxicity. In an AD brain, there is an impairment of the defense mechanism against oxidative stress due to a reduction in the concentration of glutathione. Furthermore, generation of ROS in the brain inhibits the activity of α-secretase whilst promoting the activity of γ- and β-secretase via generation of neurotoxic β-amyloid 40 and 42 [35]. These two mechanisms form a vicious cycle in the AD pathology. Another mechanism notably suggested that β-amyloid impairs mitochondrial function of neuronal cells in AD patients; therefore, promoting neuronal cell death by inducing oxidative injury in isolated mitochondria. β-amyloid impairs the antioxidative stress mechanism by lowering the expression of uncoupling proteins (UCPs) that act to promote mitochondrial uncoupling and reduce ROS generation [35]. A limited number of studies proposed that oxidative stress susceptibility is increased by overexpression of tau protein in neuronal cells. In addition, presence of transition metals including iron, zinc, and copper can react with the β-amyloid to produce hydrogen peroxide (H_2_O_2_) in the brain [34,35,36].

Fermented papaya preparation (FPP) is a popular health-promoting product which owns protective properties against free radicals to improve general health. Meanwhile, fermented papaya preparation (FPP) exerted neuroprotective properties against copper induced neurotoxicity in Swedish mutant human APP (APPsw) cells at a dose of 2.4 mg/mL by reducing 64% of ROS generation. In addition, FPP significantly reduced scavenging of superoxide anion and hydroxyl radicals and upregulation of SOD-1 enzyme. FPP exerted anti-apoptotic effect and attenuated pro-apoptotic Bax gene expression, upregulated BCL level, and maintained calcium homeostasis, leading to improvement of neuronal cell survival and AD condition. Administration of 2.4 mg/mL FPP inhibited up-regulation of expression of iNOS, nNOS, and NO by about 43%, 71%, and 40%. Moreover, treatment with 2.4 mg/mL FPP lowered the secretion of Aβ peptide by 30.6% [37].

FPP significantly reduced the 8-hydroxy2′-deoxyguanosine (8-OHdG) level in AD patients treated with FPP at a dose of 4.5 g/day for 6 months. During the study period, no neurotrophic drugs were administered to the study participants; therefore, proving the value of the *Carica papaya* plant in improving AD. The proposed mechanisms of action by FPP include decreased peroxidation of lipids, aluminum and iron induced neuronal toxicity and free radicals’ production [38]. Overall, FPP showed promising anti-Alzheimer’s disease in cell-based model and human trial. Studies including discovery of novel phytochemicals, safety profile, and efficacy warrants future investigation.

### 3.4. Periodontal Disease

Periodontal disease is an infection on the tissue that supports the tooth, which is closely related to oxidative stress. Several examples of periodontal diseases include gingival inflammation, chronic periodontitis, aggressive periodontitis, necrotizing periodontal disease and periodontal associated lesion. These conditions can happen to anyone ranging from a juvenile to an adult [39]. When the integrity of tissues supporting the tooth is compromised, the immune response of the host is triggered secondary to pathogen invasion and eventually led to inflammation. Periodontal inflammation can be augmented by excessive ROS and leukocytes and damaging the periodontal tissues. Likewise, periodontal tissue injury also occurs when there is a disruption in the redox equilibrium, due to either over-generation of ROS or diminished antioxidant enzymes, including GPx, CAT, and SOD, which defend against oxidative stress. ROS plays a role in activating signaling pathways, such as NLRP3 inflammasomes, NF-*κ*B, and JNK, which eventually lead to inflammation and cell death [40]. Human trial has shown that *Carica papaya* leaf extract significantly alleviated gingival bleeding and inflammation [41].

Kharaeva and colleagues reported that the standardized fermented papaya gel (SFPG) application at 7 g/day for 10 consecutive days significantly reduced gingival inflammation and bleeding in participants by decreasing nitrate (NO_3_^−^) and nitrite level (NO_2_^−^), and regulating the level of inflammatory cytokines. Reduced NO_3_^−^ and NO_2_^−^ attenuated production of peroxynitrite and oxidative stress generation. Furthermore, the antioxidant effect was reported to last as long as 35 days after stopping SFPG application. Interestingly, SFPG was able to augment bacterial killing by impeding activation of bacterial catalase and eventually prevent infection at the periodontal sites [42].

Studies have also shown the protective effects of *Carica papaya* leaf extract dentifrice on interdental gingival bleeding. Participants who used dentifrice containing *Carica papaya* leaf extract demonstrated a significant decrease in the gingival bleeding and inflammation especially in advanced (>70%) gingival bleeding cases. This result could be attributed to the high phenolic content of *Carica papaya* leaf extract that possess antioxidant properties. A study revealed that *Carica papaya* leaf extract exerted anti-inflammatory action by decreasing TNF-α [41].

### 3.5. Skin Aging

Skin aging is characterised by extracellular matrix (ECM) degradation in which human skin naturally becomes drier, thinner, unevenly pigmented, and wrinkled, as a human being ages, due to the inevitable intrinsic aging factors. Extrinsic aging factors are avoidable, in which both factors may synergize and lead to premature skin aging. ROS is known to be the culprit of skin aging by contributing to oxidative stress and inflammation. Photoaging is a process that produces ROS, which eventually leads to augmented ECM turnover and degradation. Although not fully deleterious to the cells, excessive ROS can oxidise skin proteins and lipids leading to roughen the skin by altering the function of the skin barrier and further stimulate wrinkle formation [43].

In addition, ultraviolet (UV) and UV-generated ROS hasten aging via activation of mitogen-activated protein kinase (MAPK), p38, Jun N-terminal kinase (JNK), extracellular-signal-regulated kinase (ERK), recruitment of c-Fos, and c-Jun, as well as increased expression of activator protein-1 (AP-1) and nuclear factor kappa B (NF-κB). AP-1 is known to lower transforming growth factor-beta (TGF-β), which is responsible for collagen production and induce expression of matrix metalloproteinase (MMP) 1, 3, and 9 in keratinocytes, and fibroblast leading to the disruption and loss of ECM components (collagen and elastic fibers) [43]. UV and ROS causes the skin to be in the state of “sunburn” (erythema). This further stimulates production of advanced glycation end products (AGEs). Activation of receptor for AGEs (RAGE) increases NF-κB activation, thereby upregulates pro-inflammatory gene transcriptions and RAGE leading to a vicious inflammatory state cycle characterised by elevated PGE2 synthesis [43,44].

Furthermore, ROS induces melanogenesis by increasing the number of tyrosinase-related protein 1 (TYRP-1) and tyrosinase, which are both known as melanogenic factors resulting in skin pigmentation [44]. In addition, UV radiation induced greater amounts of oxidised lipids, triglyceride hydroperoxides, and cholesterol hydroperoxides generation, leading to increased sebum secretion. This condition in turn promotes the formation of acne vulgaris by *Propionibacterium acnes* (*P. acnes*). *P. acnes* infects skin cells and will further induce the production of free oxygen radicals that eventually lead to the formation of inflammatory lesions [44].

In past decades, research on strategies against skin aging attracted a great attention of researchers. For instance, *Carica papaya* is a potential candidate to be exploited for its anti-skin aging specialty, owing to its antioxidant and anti-inflammatory activities. Table 3 shows anti-skin aging activities of *Carica papaya* extracts. An in vitro study by Jarisarapurin and colleagues focused on unripe *Carica papaya* fruit extract against skin aging related endothelial oxidative stress [45]. It was proposed that activated endothelial cells contributed to a low-grade inflammatory state and the generation of oxidative stress. As a result of this unfavorable microenvironment, MMP-1 expression in dermal fibroblasts was induced leading to a significant loss of type I collagen, and accelerated ECM degradation [46]. The study demonstrated the ability of unripe *Carica papaya* fruit extract to inhibit H_2_O_2_-induced endothelial cell death at concentrations ranging from 100 to 1000 μg/mL. It was found to exert its effect via modulating intracellular stress and antioxidant defenses in endothelial cells. The mechanisms were consisted of a dose-dependent ROS scavenging effect and NF-κB attenuation, upregulation of SOD and CAT activities, and prevention of H_2_O_2_-induced Nrf2 over activation. The study further explained that, although activation of antioxidant defenses was prompted by uncoupling of the Nrf2/Keap1 complex, followed by translocation of Nrf2 into the nucleus, the early (or over activation) of Nrf2 induced by oxidative stress can lead to depletion of endogenous antioxidants. The consequence of depletion of natural antioxidants produced by skin cells may promote skin aging. Therefore, the restraining properties of unripe *Carica papaya* on NF-kB elevation and Nrf2 dysregulation were proposed to be beneficial in maintaining redox homeostasis, thereby delaying skin aging [45].

A recent study by Seo and colleagues investigated the anti-aging mechanisms of *Carica papaya* leaf ethanol extract on UVB-irradiated human dermal fibroblast cells in vitro. At concentrations ranging from 10 to 250 μg/mL, the extract demonstrated radical scavenging and ROS suppressing action in a dose-dependent manner. At concentrations of 1 to 50 μg/mL, the extract was shown to enhance synthesis and attenuate degradation of type I procollagen in UVB-irradiated fibroblasts, increment in TGF-β1, and reduction in MMPs (MMP-1 and MMP-3) generation. Interestingly, Seo and colleagues further evaluated that the leaf extract possessed reversal action on UVB-induced AP activation at mRNA level via downregulating MAPK activation and protein phosphorylation of c-Fos and c-Jun. The effect of *Carica papaya* leaf extract on MAPK was proposed to act mainly on p38, showing 82% inhibition against p38 phosphorylation, followed by ERK and JNK. The extract demonstrated to acquire anti-inflammatory action by depleting production of cytokines, such as IL-6. Wrinkles formation induced by sun exposure as a result of erythema and diminished Type I collagen in skin. The ROS-conquering mechanisms and collagen synthesis promoting effects were described by Seo and colleagues, lending support on the potential use of *Carica papaya* leaf extract against skin aging [47].

A human trial by Bertuccelli and colleagues revealed that sublingual FPP 4.5 g sachet twice daily lowered biomarkers of skin aging. While both treatments attenuated skin MDA level, FPP showed superior anti-aging effects than antioxidant cocktails [45]. In addition, FPP elevated levels of SOD, NO, aquaporin-3 (AQP-3), and down-modulation of pro-aging cyclophilin-A (CyPA) and CD147 genes. The study proposed that the regulating effects of FPP on AQP-3 and pro-aging factors were crucial for significant improvement in skin health [48].

The potentiality of *Carica papaya* being formulated as cosmetic products was demonstrated by Saini and colleagues, as the ideal oil-in-water *Carica papaya* fruit cream prepared was uniform, stable, and had a shiny and smooth texture. This study further proved ROS suppression as the main mechanism of *Carica papaya* fruit against anti-aging, in which the 5% cream was potent, owing reducing power against H_2_O_2_ free radicals [49].

Flavonoids and phenolic acids were found in *Carica papaya* leaf and fruit extracts [50,51]. Flavonoids in *Carica papaya* are mainly kaempferol, myricetin, quercetin, and their glycosides, phenolic acids, such as caffeic acid and ferulic acid, are the key ROS suppressors and antioxidant that displayed radical scavenging and metal chelating potential [50,52]. Caffeic acid and rutin were detected and proposed to be the main anti-skin aging components. Both phytochemicals were reported to downregulate MMPs expression and photoprotective against collagen degradation. Caffeic acid mitigated skin erythema via inhibitory action towards NF-κB and AP-1 signaling [48]. The ability of caffeic acid in film formulation to permeate and retentate in epidermis (stratum corneum) and dermis layer enhance its efficacy [50,53]. Besides, the anti-skin aging role of rutin was supported by a human trial, which showed enhanced skin elasticity and less wrinkles in individuals treated with rutin-containing cream. The findings of elevated type I collagen via lowering MMP expression and potent ROS scavenging in human dermal fibroblast cells further supported the anti-skin aging of *Carica papaya* chemical constituents [48,54].

Albeit several mechanisms were compiled and proposed, however, studies regarding the *Carica papaya* anti-skin aging effect were scarce. More evidence regarding various parts, therapeutic range, and the relevant phytochemicals of *Carica papaya* on skin aging are needed to ensure their efficacy.

### 3.6. Wound Healing

Wound healing is rather complex and well-coordinated with involvement of several stages of cellular responses, including inflammation, proliferation, and remodeling. The duration of each phase usually ranges from 1 to 4 days, 5 to 10 days, and 11 days onwards. Characterisation for each phase includes presence of leukocytes, angiogenesis, protein synthesis and deposition, epithelialization, wound contraction, and scar formation [55]. These processes can be altered by the presence of oxidative stress [56]. The efficiency of the wound healing process decreases with advancing age [57]. Oxidative stress can alter the speed of wound recovery as it depends on the amount of ROS present at the wound site. Although minimal ROS prevents infection, excessive ROS is known cytotoxic to fibroblasts and reduce flexibility of skin lipids. In addition, it also causes impairment to lipids, DNA, proteins, and cellular membranes, and subsequently, severely damages the tissue and promotes inflammation [56].

In the case of chronic wounds or impaired wounds, ROS production is excessive in response to NADPH oxidase (NOX) activation in macrophages and neutrophils during the inflammatory phase of the wound healing process, contributing to high oxidative stress that leads to the wound remaining not healed. Thereby, extracts and phytochemicals with great strength of antioxidative properties are beneficial in wound healing [58,59]. Another key factor for wound healing is the extent of inflammation level at different stages of healing. Inflammation is essential to prevent infection, stimulate angiogenesis, and matrix deposition via secretion of cytokines and angiogenic factors at the early stage of wound healing. However, excessive or prolonged pathological inflammation causes delayed wound healing and fibrosis. Hence, inflammation at certain phases is deemed crucial for wound healing, but not throughout the entire healing process [55,60].

It is known that oxidative stress plays a vital role in wound healing. Table 4 shows wound healing activities of *Carica papaya* extracts. The protective action of aqueous extract *Carica papaya* seeds against oxidative stress-induced apoptosis in human skin fibroblast further supported its role in wound healing. An extensive mechanistic study conducted found potent antioxidant action of the papaya extract against H_2_O_2_-induced oxidative stress specifically on fibroblast cells was activated via radical scavenging, reduction of calcium ions influx into cytoplasm, reversal of oxidative stress-induced mitochondrial dysfunction, and maintaining oxidative balance inside the cells [61]. Mikhal and colleagues showed that FPP possesses antioxidative stress and anti-inflammation activities. FPP inhibited superoxide (IC_50_ = 5 mg/mL), hydroxyl radicals (IC_50_ = 1.1 mg/mL), and total ROS (IC_50_ =2 mg/mL) in blood, as well as reduction in myeloperoxidase (MPO) and radical generation at wound sites in vivo [62].

In addition, topical application of 5 mg/mL *Carica papaya* fruit extract enhanced wound healing by exerting effect on regulation antioxidant enzymes, inflammation, and arginine metabolism. The addition of an antioxidant, selenium to the regimen, further shortened the time for wound healing significantly and, hence, confirmed the mechanisms. Antioxidative stress related mechanisms include inhibition of lipid peroxidation, lower MDA level and enhanced expression of SOD, CAT, and GPx. *Carica papaya* fruit extract reduces inflammation associated with oxidative damage through upregulation of antioxidant enzymes, arginine metabolism, and cyclooxygenase specific inhibition in an excision wound model. The extract demonstrated an attenuated inflammatory state, increased collagen synthesis and vascularization at wound site. Transforming growth factor-beta (TGF-β), a cytokine that generates fibroblast recruitment was high at the inflammatory phase and reduced at the repairing phase. While expression of vascular endothelial growth factor A (VEGFA), an angiogenic factor was increased throughout the wound healing process. The further study showed that addition of selenium to the papaya fruit extract synergistically upregulated TGF-β and VEGFA resulting in a significant acceleration in the wound healing process [63,64].

An oral FPP supplementation at a dose of 0.2 g/kg body weight for 8 weeks was found to enhance diabetic wound closure via improved macrophages respiratory-burst function and iNOS production. Diabetic wounds are hard-to-heal due to being prone to infections as a result of compromised NO at the wound site. Another reason was the antibacterial effect of macrophages via NOX was downregulated by hyperglycemia, consequently, respiratory burst dysfunction was seen in diabetic patients. FPP was shown to reverse these conditions. Similar to previous report by Nafiu and colleagues, FPP supplement showed an increase in VEGFA expression, deemed as a crucial regulator in current scenario [65].

Dickerson and colleagues further examined the diabetic wound healing effect of FPP on type II diabetes mellitus patients. The participants were given oral FPP (9 g/day for 6 weeks), and showed that NADPH and cellular ATP level increased in human monocytic THP-1 cells treated with FPP. Besides, FPP also exhibited higher oxygen usage and mitochondrial membrane potential on monocytic cells, which further revealed its capability to correct the respiratory burst function, enhancing the defense mechanisms against pathogens in diabetics. FPP upregulated the mRNA expression of Rac2, which was essential for NOX activation and eventually enhancing respiratory burst in macrophages [66,67].

Meanwhile, Indran and colleagues investigated the protective effect of *Carica papaya* leaf aqueous extract against alcohol-induced hemorrhagic lesions. Pretreatment with 500 mg/kg leaf extract significantly reduced gastric ulcer index via reducing lipid peroxidation, MDA levels, and improving GPx activity at gastric mucosa. The study showed the radical scavenging activity, which might be contributed by polyphenols within leaf extract. It was suggesting that the alkaline content of the extract and its ability to neutralize stomach acidity, thereby protecting stomach against gastric ulcer [68]. The concepts were further supported by in vivo studies evaluating different parts of *Carica papaya* on incised, burned, and diabetic wounds respectively. The recent findings show that *Carica papaya* fruit and seed extracts demonstrated dose-dependent increment in hydroxyproline, fibrillation, epithelial thickness, shortened wound contraction, and epithelialization time [69,70,71,72].

**Table 4:** Wound healing activities of *Carica papaya.*

Part of the Plant	Extract	Type of Experiment	Results	Reference
Seed	Aqueous extract	In vitro cytoprotective assay	Aqueous extract of papaya seeds at 1mg/mL showed cytoprotective against H_2_O_2_ induced cell toxicity.	[61]
		Cell apoptosis assay	Aqueous extract of papaya seeds at a concentration of 1 mg/mL inhibited H_2_O_2_ induced apoptosis by approximately 30%.	
		MMP and Cytochrome C assay	Seed extract at 1 mg/mL inhibited oxidative stress-induced cell apoptosis, reduced mitochondrial dysfunction and impeded release of cytochrome C.	
		Western blot analysis	1 mg/mL of seed extract decreased overexpression of HSP-70 in fibroblasts.	
-	Fermented papaya (Biorex)	In vitro HRBC model	Biorex inhibited superoxide (IC_50_ = 5 mg/mL), hydroxyl radicals (IC_50_ = 1.1 mg/mL), and total ROS (IC_50_ = 2 mg/mL) in human red blood cells.	[62]
		In vitro animal model	Biorex (1–5 mg/mL) decreased the elevated radical generation in rats with burn trauma. Biorex reduced local inflammation and catalase activity.	
Unripe pulp	Papaya extract +/− Selenium	In vitro animal model	Papaya extract alone (PE) or with selenium (PES) enhanced wound closure in rats. Both PE and PES augmented SOD, CAT, and GPx activities. PE with selenium ameliorated oxidative damage at the wound site. PE enhanced wound healing via attenuating excessive inflammation, reduced COX-2, and MPO enzyme activity. PE and PES increased NO content by increasing iNOS, stimulating collagen deposition and angiogenesis. PE suppressed arginase activity during wound healing as indicated by decreased wound urea content.	[63]
Unripe papaya pulp	Papaya aqueous extract. Or Papaya PBS extract + Selenium	In vitro animal model	Total protein content (95.14 ± 1.15 mg/g tissue) in wound tissue was significantly higher in rats treated with PES at a dose 5 mg/mL twice daily for papaya and 0.5 μg/20 mL for selenium. Rats treated with PES demonstrated elevation in wound hydroxyproline (*55.15 ± 1.06 μg/mg), hexuronic acid (*60.84 ± 6.08 mg/g), and hexosamine (*35.23 ± 4.95 mg/g) contents. Overall reduced in migration of polymorphonuclear monocytes and increased fibroblast recruitment at wound sites. PE enhanced collagen synthesis and vascularization. Time required for wound closure was shortened, indicated by earlier increment in VEGFA and TGF-β1 expression.	[64]
-	FPP	In vitro animal model	FPP at a dose of 200 mg/kg s improved wound closure via increasing ROS (superoxides) production by macrophages at wound site and promoting NO production at ~60%. Increased NO and ROS to support redox signaling and angiogenesis. FPP increased CD68, VEGF transcription, macrophages recruitment to wound site and promoted optimal angiogenesis environment.	[65]
Leaf	Aqueous extract	In vitro animal model	Aqueous extract of papaya leaves at a dose of 500 mg/kg protected the stomach from absolute ethanol induced injury. Aqueous extract decreased MDA levels by 0.031 μmol/L and increased GPx by 0.246 U/mg protein.	[68]
Fruit	Aqueous extract	In vitro animal model	Aqueous extract of papaya fruit significantly shrank the wound area at 100 mg/kg by 77% by increasing epithelialization rate, weight of dry and wet granulation tissues and promoting enzymatic debridement of wound. Aqueous extract-treated wound showed rapid collagen turnover and accumulation that enhanced wound healing.	[69]
Tree	Dried latex incorporated into hydrogel	In vitro animal model	Topical application of the dried latex-containing hydrogel (1–2.5%) increased hydroxyproline content. Significant wound contraction after application of this hydrogel day 12 at concentration of 2.5% and on day 20 at both concentrations of 1.0% and 2.5%.	[70]
Seed	Ethanol extract	In vitro animal model	Ethanol extract of papaya seeds at a dose of 50 mg/kg significantly reduced wound area by 88.96%. Ethanol extract produces well-organized collagen deposition and significant fibroblast activity.	[71]

Abbreviation: MMP, matrix metalloproteinase; HRBC, human red blood cell; HSP-70, heat shock protein 70; NO, nitric oxide; ROS, reactive oxygen species; COX-2, cyclooxygenase-2; MPO, myeloperoxidase; VEGFA, vascular endothelial growth factor A; TGF, transforming growth factor; FPP, fermented papaya preparation; MDA, serum malondialdehyde.

Cysteine endopeptidases including papain and chymopapain showed wound healing activity that can be attributed to their proteolytic wound debridement and antibacterial effects [63,64,70,72]. This was established by an in vivo study using papain-based wound cleanser. The cleanser was formulated with 5 g of papain and α-tocopherol. The results showed superior collagen deposition and least fluid exudates compared to betadine cleanser leading to eschar reduction and quicker epithelialization [73]. Safety of *Carica papaya* extracts and dressings is of less concern as it is traditionally used to treat wounds and certain skin conditions [74]. Several studies further assured its safety to be used [61,67,71]. In addition, papaya dressing was safe to be used and compatible in hydrogel formulation [70,75].

### 3.7. Cancers

Cancer is a prevailing topic and there is no absolute cure to date for various types of cancers. ROS generation as a result of metabolic reactions in the mitochondria plays a role in both initiation and potentially elimination of cancers. With a low amount of ROS that is tolerable by the body cells, the progression of cancer could occur through either promoting genomic DNA alterations or DNA damage that alters the normal physiological signaling pathways. For instance, mitogen-activated protein kinase (MAPK) activation, c-Jun N-terminal kinase (JNK), extracellular signal-regulated kinase (ERK) phosphorylation, and cyclin D1 expression are correlated to cancer progression and survival [76]. In the normal healthy cells, a significantly high level of ROS can lead to cellular damage and eventually cell death [77]. However, cancer cells generally have a higher resistance to oxidative stress than normal cells to allow for uncontrolled proliferation and to compensate for the survival of cancer cells during metastasis from their site of origin [76]. However, increasing ROS to a specific threshold level, specifically for cancer cells is proven to attenuate cancer cell growth and progression.

Several studies showed the correlation of microRNAs and oxidative stress in the progression of cancer. The recent advancement of genomic studies has showcased the presence of certain groups of microRNAs may promote cancer cell proliferation and progression. For example, there is an overexpression of miR-210 detected in hepatocellular and breast carcinoma under hypoxia. miR-210 acts to regulate the ROS production and mitochondrial function by promoting cancer cell adaptation, survival, and proliferation [77]. It also suggested that ROS is able to induce carcinogenesis by induced mutations in the tumour-suppressor gene in the normal skin cells leading to a transformation of normal cell into cancerous cells by halting the initiation of programmed cell death. An example of this mutation is seen in the alteration of a guanine in the p53 gene through oxidative mechanisms in basal cell and squamous cell carcinoma [43].

DNA damage is pivotal in cancer formation. A study proposed that FPP was capable of impeding DNA fragmentation induced by free radicals and H_2_O_2_-induced DNA damage at a dose of 100 μg/mL [16]. In addition, the aqueous extract of *Carica papaya* fruit suppressed proliferation of human breast epithelial cancer (MCF-7) cells in vitro. The aqueous extract of *Carica papaya* showed significant anti-proliferation activity (~53%) in MCF-7 cells at a dose of 4% *v*/*v* after 72 h treatment. The anti-cancer activity of FPP might be attributed to the mechanisms including triggering cell signaling to induce apoptosis [78]. An in vitro study showed that the aqueous extract of *Carica papaya* leaves showed antiproliferation activity of MCF-7 at a IC_50_ of 1.31 mg/mL and induced apoptosis of MCF-7 cells at 22.5% with a dose of 0.65 mg/mL [79].

*Carica papaya* enriched with phytochemicals, including flavonoids, has been found to possess chemopreventive properties. The mechanisms of action underlying the chemoprevention effects include activating tumour-suppressor genes, deactivating oncogene products transcriptionally, decreasing oxidative damage via acting as free radical scavengers and impeding the commencement of lipoxygenase reaction by chelating with ROS-generating agents. For example, the benzene fraction of aqueous extract of *Carica papaya* showed chemoprotective effects in benzo(a)pyrene and 7,12-dimethyl benz(a)anthracene -induced carcinogenic animal models. It was reported that a significant reduction of lung adenomas (>50%) at a treatment dose of 1 g/kg body weight. In addition, a significant reduction in skin papillomagenesis incidence at 64.20% was compared with tumour incidence in the control group. It was suggested that those flavonoids contained within different parts of the *Carica papaya* plant act via multi-signaling networks as the viable chemoprevention agents [80].

An in vivo study reported that FPP at a concentration of 500 mg/kg significantly elevated antioxidant enzymes, including GPx (66.1%), SOD (20%), and CAT (81%). Furthermore, FPP was also capable of preventing DNA structural damage possibly induced by free radicals and genotoxins [81]. This was supported by another study suggesting that *Carica papaya* peel extract significantly increased glutathione (GSH), while decreasing MDA and ROS production. Thus, preventing DNA damage and induction of colonic carcinogenesis azoxymethane treated group [82]. Another study by Mukami and colleagues revealed that orally administration of FPP at 450 mg/kg showed complete disappearance of the tumours in a radiation-induced leukemia mice model [83]. Overall, several research groups revealed the anticancer properties of papaya extracts. Further studies are needed to standardize the extract for quality control of the efficacy, and discover novel compounds, owing to the anticancer activities.

## 4. Conclusions

To summarize, the *Carica papaya* counteracts oxidative stress via its potent antioxidant properties. Therefore, it can be incorporated into nutraceuticals or conventional medications to be used as a potential preventative or treatment option for various health conditions. The antioxidant properties of the *Carica papaya* plant might be attributed to the various chemical constituents that the plant contains, including caffeic acid, myricetin, quercetin, rutin, α-tocopherol, papain, BiTC, kaempferol steroids, alkaloids, and saponins.

There is no doubt that emerging evidence has proven the potential of *Carica papaya* as a natural resource that can be exploited as a medicinal product. However, more safety data are needed to justify its use in different medical conditions. Many plants—although exerting therapeutic benefits, having been used traditionally for diseases since the ancient times—are potentially cytotoxic [7,84]. The acute toxicity study of the *Carica papaya* leaf extract revealed that there were no significant toxic effects of *Carica papaya* leaf extract at the concentration up to 2 g/kg of body weight, which corresponded to 14 times the dose incorporated in traditional medication. Moreover, it was also suggested that any concentration of *Carica papaya* leaf extract below 2 g/kg of body weight posed no significant toxicity and adverse effects when administered orally for a 14-day interval [85,86]. In terms of the *Carica papaya* extract with different methods of extraction, namely ethanol and aqueous extract, it might possess different safety profiles, owing to the extractive chemical constituents. For example, the ethanol extract might be more nephrotoxic and hepatotoxic than the aqueous extract in the Wistar rats model at 1 g/kg of body weight concentration [87]. However, all these studies suggest that more extensive evaluation studies pertaining the cytotoxicity profile of oral administration of the *Carica papaya* extract are needed to further validate the safety for consumption.

It was also suggested that the medicinal properties of the *Carica papaya* plant can be attributed to other mechanisms of action. Several studies have suggested that *Carica papaya* extract exerted antimicrobial properties that aided in wound recovery [69,71]. Therefore, more studies should be done in order to unravel the benefits of the *Carica* *papaya*.

## Figures and Tables

**Table 1:** Anti-inflammation activities of *Carica papaya.*

Part of the Plant	Extract	Type of Experiment	Results	Reference
Seed	Aqueous extract	In vitro cell free model In vitro HRBC assay	Aqueous extract of papaya seeds at 20 μg/mL decreased NO radical by 69.4%, comparable to ascorbic acid.	[15]
			Aqueous extract of papaya seeds at 150 μg/mL inhibited the release of lysosomal enzyme by 22.7%.	
Leaf	Leaf extract	In vitro animal model	Leaf extract at 1.32 μg/mL enhanced adaptive immune response by upregulated TLR-7 and TLR-9 expressions.	[17]
Fruit (ripe, unripe fruit, peel, seed, pulp)	Aqueous extract	In vitro ROS assay	Unripe peel (69.7%) and seed (79.1%) extract at 2mg/mL showed ROS scavenging activity of 69.7 and 79.1% at 2 mg dry weight/mL.	[18]
		In vitro antioxidant enzyme assay	Aqueous extract of papaya unripe peel at 2 mg dry weight/mL increased SOD activity by 21.9%.	
		In vitro protein carbonyl assay	Aqueous extract reduced oxidative damage by lowered protein carbonyl production for ripe seed (60.4%) and ripe peel (57.6%) extract at 0.1 and 2 mg/mL.	
		In vitro inflammatory cytokines assay	The extracts augmented IL-10 levels at a low concentration of 0.1 mg/mL. Seed extracts exerted highest increment in IL-10 secretory level (+140.1%), followed by peel and pulp extracts. Seed extracts at 0.1 mg dry weight/mL exerted increment in IL-10 secretory level (+140.1%).	
			Aqueous extract of papaya seeds at 2 mg dry weight/mL down regulated IL-6 by 37.8%.	
			Unripe extracts at 2 mg/mL showed inhibitory activity against TNF-α with 71.2% for pulp extract, 62.7% for peel and 65.3% for seed extract.	
Fruit (Flesh)	Juice	In vitro animal model	Papaya juice downregulated the elevated serum IL-6 (217.6 vs. 28.3 pg/dL) and MDA (3.2 vs.1.4 pg/dL) in high fat diets treated rats. Papaya juice affected serum SOD of the high fat treated rat by increased serum SOD (30.41 U/L).	[19]
Leaf	Ethanol extract	In vitro animal model	Ethanol extract of papaya leaves at 200 mg/kg reduced paw edema (2.6 mm) and inhibited granuloma formation (0.2 g).	[20]

Abbreviation: NO, nitric oxide; HRBC, human red blood cell; TLR, toll-like receptors, MDA, serum malondialdehyde.

**Table 2:** Anti-diabetes activities of *Carica papaya.*

Part of the Plant	Extract	Type of Experiment	Results	Reference
Seed	Hexane extract & ethyl acetate extract	In vitro DPPH radical scavenging assay	Hexane extract possessed DPPH radical scavenging activity with IC_50_ = 41.5 mg/mL.	[27]
		In vitro TBA method	Hexane extract demonstrated TBA scavenging activity with IC_50_ = 38.2 mg/mL.	
		In vitro α-glucosidase inhibition	Hexane extract displayed α-glucosidase enzyme inhibitory activity with IC_50_ = 75.78 mg/mL.	
			Ethyl acetate extract exhibited α-glucosidase enzyme inhibitory activity with IC_50_ = 77.41 mg/mL.	
		In vitro α-amylase inhibition	Hexane extract demonstrated α-amylase inhibitory activity with IC_50_ = 76.96 mg/mL.	
			Ethyl acetate extract displayed α-amylase inhibitory activity with IC_50_ = 79.18 mg/mL.	
		In vitro FRAP assay	Ethyl acetate extract displayed FRAP inhibitory activity with IC_50_ = 38.75 mg/mL.	
		In vitro animal model	Ethyl acetate extract at 500 mg/kg/body weight significantly decreased the blood glucose level of the diabetic rats to approximately 120 mmol/L over 120 min comparable with standard drug, acarbose.	
-	FPP	In vitro analysis	FPP at concentration 50 μg/mL increased inner and outer platelet membrane fluidity, displayed by a decrease of ~0.015 r in DPH anisotropy and ~0.02 r in TMA-DPH anisotropy. FPP increased Naþ/Kþ-ATPase activity by ~0.5 μmol Pi/mg prot/h.-FPP improved platelet function in vitro and this might help preventing diabetic complications. FPP also slightly increased TAC by ~5 nmol/μL and SOD activity by ~0.5 units/μL. FPP at 50 μg/mL lowered lipid peroxidation.	[28]
-	FPP^®^	Human trial	FPP significantly improved liver sensitivity to insulin, which was indicated by decreased circulating AST and ALT. FPP scavenged NO and hydroxyl radicals and displayed an increased in total antioxidant status.	[29]
-	FPP	In vitro DPPH radical scavenging assay	FPP displayed DPPH scavenging with AA_50_ = 55.69 mg/mL.	[30]
		In vitro ABTS^+^ scavenging assay	FPP demonstrated ABTS^+^ scavenging action with AA_50_ = 14.56 mg/mL.	
		In vitro AAPH-induced lipid oxidation inhibition	FPP inhibited AAPH-induced lipid oxidation with AA_50_ = 68.06 mg/mL.	
		In vitro O_2_^−^ scavenging assay	FPP showed O_2_^−^ scavenging action with AA_50_ = 88.70 mg/mL.	
		In vitro •OH scavenging assay	FPP showed hydroxyl radical scavenging activity with AA_50_ = 4.13 mg/mL.	
		Human trial	FPP at a dose of 6g/day showed an increase of 4.9% and 5.7% in TAS for male and female respectively after 14-week consumption at 6g/day. FPP decreased protein carbonyl level by 1.9% in males and 9.7% in females after a 14-week FPP ingestion. FPP delayed red blood cell hemolysis.	
Seed, flesh and peel of unripe fruit	Aqueous extract	In vitro α-amylase inhibition In vitro α-glucosidase inhibition In vitro lipid peroxidation assay In vitro NO scavenging assay	Aqueous extract inhibited α-glucosidase and α-amylase activities with IC_50_ of 1.76 mg/mL and IC_50_: 0.87 mg/mL. At a concentration of 7.5 mg/mL, the extract also displayed the highest NO radical scavenging activity (52.5%).	[31]
Leaf	Chloroform extract	In vitro animal model	The chloroform extract at a dose of 31 mg/kg/day significantly increased islet area by 16,842.2 μm^2^ by stimulating regeneration of β-cells of islet of Langerhans.	[32]
			The extract successfully decreased fasting glucose levels by 222.3 mg/dL in diabetic group in vivo.	
Leaf	Aqueous extract	In vitro animal model	Aqueous extract at a dose of 3 g/100 mL decreased blood glucose levels in diabetic rats by 184 mg/dL. Aqueous extract at a dose of 1.5 g/100 mL preserved Islet cell size in diabetic rats. Aqueous extract increased NO levels by 17.39 μM and hence reduced ROS production.	[33]

Abbreviation: DPPH, 2,2-diphenyl-1-picryl-hydrazyl-hydrate; TBA, thiobarbituric acid; FPP, fermented papaya preparation; TAC, total antioxidant capacity; SOD, superoxide dismutase; AST, aspartate transaminase; ALT, alanine aminotransferase; NO, nitric oxide. Footnote: IC_50_ = concentration needed for 50% inhibition; AA_50_ = concentration needed to achieve 50% antioxidant activity.

**Table 3:** Anti-skin aging of *Carica papaya.*

Part of the Plant	Extract	Type of Experiment	Results	Reference
Unripe papaya juice		In vitro antioxidant enzyme assays	Papaya juice at 1 mg/mL enhanced SOD (49%) and CAT (40.5%) activities.	[45]
		Western blot analysis	Papaya juice at 1 mg/mL restrained NF-*κ*B translocation to nuclei and downregulated Nrf2 levels.	
Leaf	Ethanol extract	In vitro DPPH assay	Ethanol extract at 250 μg/mL showed ROS scavenging effect at 60%.	[47]
		In vitro DCFH-DA assay	Ethanol extract at 50 μg/mL showed potent suppressing action towards UVB-induced ROS production (60%).	
		In vitro MMPs and inflammatory cytokines production	Ethanol extract at 50 μg/mL of *Carica papaya* leaves enhances synthesis and prevents degradation of type I collagen via upregulating TGF-β1 and down-regulating MMP-1 (34% at 50 μg/mL), MMP-3, and IL-6 generation. Ethanol extract at 50 μg/mL of *Carica papaya* leaves reduced mRNA level of MMP-1 (56.8%) and type I procollagen (288.8%).	
		Western blotting assay	Ethanol extract at 50 µg/mL showed AP-1 activation via down-regulating c-Fos (89%) and c-Jun (44%) phosphorylation. Ethanol extract at 50 µg/mL attenuated MAPK activation, and p38 phosphorylation (82%), followed by ERK, and JNK phosphorylation.	
	FPP	Double-blinded RCT	FPP at a dose of 4.5 g showed anti-skin aging by demonstrating overall higher skin moisturization, elasticity, and surface evenness. FPP at a 4.5 g inhibited MDA production and up modulation of AQP-3, enhanced SOD and NO levels in FPP-treated group. FPP at 4.5 g downregulated pro-aging factors (CyPA and CD147 genes) suggesting to reduce risk of skin carcinogenesis.	[48]

Abbreviation: DPPH, 2,2-diphenyl-1-picryl-hydrazyl-hydrate; DCFH-DA, dichloro-dihydro-fluorescein diacetate; SOD, superoxide dismutase; MMP, matrix metalloproteinase; RCT, randomized controlled trial, MDA, serum malondialdehyde.

## Data Availability

Not applicable.

## References

[1] Sies H. (2015). Oxidative stress: A concept in redox biology and medicine. Redox Biol..

[2] Pizzino G., Irrera N., Cucinotta M., Pallio G., Mannino F., Arcoraci V., Squadrito F., Altavilla D., Bitto A. (2017). Oxidative Stress: Harms and Benefits for Human Health. Oxidative Med. Cell. Longev..

[3] Aradhya M.K., Manshardt R.M., Zee F., Morden C.W. (1999). A phylogenetic analysis of the genus Carica, L. (Caricaceae) based on restriction fragment length variation in a cpDNA intergenic spacer region. Genet. Resour. Crop Evol..

[4] Yogiraj V., Goyal P., Chauhan C.S., Goyal A., Vyas B. (2014). *Carica papaya* Linn: An overview. Int. J. Herb. Med..

[5] Yap J.Y., Hii C.L., Ong S.P., Lim K.H., Abas F., Pin K.Y. (2020). Effects of drying on total polyphenols content and antioxidant properties of *Carica papaya* leaves. J. Sci. Food Agric..

[6] Fei X., Yuan W., Zhao Y., Wang H., Bai S., Huang Q. (2018). Papain Ameliorates the MPAs Formation-Mediated Activation of Monocytes by Inhibiting Cox-2 Expression via Regulating the MAPKs and PI3K/Akt Signal Pathway. BioMed Res. Int..

[7] Silva C.R.d., Oliveira M.B.N., Motta E.S., Almeida G.S.d., Varanda L.L., Pádula M.d., Leitão A.C., Caldeira-de-Araújo A. (2010). Genotoxic and Cytotoxic Safety Evaluation of Papain (*Carica papaya* L.) Using In Vitro Assays. J. Biomed. Biotechnol..

[8] Park M.J., Bae Y.S. (2016). Fermented Acanthopanax koreanum Root Extract Reduces UVB- and H2O2-Induced Senescence in Human Skin Fibroblast Cells. J. Microbiol. Biotechnol..

[9] Chen L., Deng H., Cui H., Fang J., Zuo Z., Deng J., Li Y., Wang X., Zhao L. (2017). Inflammatory responses and inflammation-associated diseases in organs. Oncotarget.

[10] Luster A.D. (2002). The role of chemokines in linking innate and adaptive immunity. Curr. Opin. Immunol..

[11] Morgan M.J., Liu Z.-g. (2011). Crosstalk of reactive oxygen species and NF-κB signaling. Cell Res..

[12] Hussain T., Tan B., Yin Y., Blachier F., Tossou M.C.B., Rahu N. (2016). Oxidative Stress and Inflammation: What Polyphenols Can Do for Us?. Oxidative Med. Cell. Longev..

[13] Kanda Y., Osaki M., Okada F. (2017). Chemopreventive Strategies for Inflammation-Related Carcinogenesis: Current Status and Future Direction. Int. J. Mol. Sci..

[14] Chatterjee S., Dziubla T., Butterfield D.A. (2016). Chapter Two—Oxidative Stress, Inflammation, and Disease. Oxidative Stress and Biomaterials.

[15] Wijesooriya A., Deraniyagala S., Hettiarachchi C. (2019). Antioxidant, Anti-Inflammatory and Antibacterial Activities of the Seeds of A Sri Lankan Variety of *Carica papaya*. Biomed. Pharmacol. J..

[16] Aruoma O.I., Colognato R., Fontana I., Gartlon J., Migliore L., Koike K., Coecke S., Lamy E., Mersch-Sundermann V., Laurenza I. (2006). Molecular effects of fermented papaya preparation on oxidative damage, MAP Kinase activation and modulation of the benzo[a]pyrene mediated genotoxicity. Biofactors.

[17] Zuhrotun Nisa F., Astuti M., Mubarika Haryana S., Murdiati A. (2020). Effect of Papaya Leaves (*Carica papaya* L.) Extract on Immune Response (TLR-7, TLR-9) and Inflammation (COX-2) in Rats Induces DMBA (7,12-Dimethylbenz[a]antrasen). Pak. J. Biol. Sci..

[18] Somanah J., Bourdon E., Bahorun T. (2017). Extracts of Mauritian *Carica papaya* (var. solo) protect SW872 and HepG2 cells against hydrogen peroxide induced oxidative stress. J. Food Sci. Technol..

[19] Od-Ek P., Deenin W., Malakul W., Phoungpetchara I., Tunsophon S. (2020). Anti-obesity effect of *Carica papaya* in high-fat diet fed rats. Biomed. Rep..

[20] Owoyele B.V., Adebukola O.M., Funmilayo A.A., Soladoye A.O. (2008). Anti-inflammatory activities of ethanolic extract of *Carica papaya* leaves. Inflammopharmacology.

[21] Amazu L.U., Azikiwe C.C.A., Njoku C.J., Osuala F.N., Nwosu P.J.C., Ajugwo A.O., Enye J.C. (2010). Antiinflammatory activity of the methanolic extract of the seeds of *Carica papaya* in experimental animals. Asian Pac. J. Trop. Med..

[22] Ahmed M., Ramabhimalah S. (2012). Anti-Inflammatory Activity of Aqueous Extract of *Carica papaya* Seeds in Albino Rats. Biomed. Pharmacol. J..

[23] Skyler J.S., Bakris G.L., Bonifacio E., Darsow T., Eckel R.H., Groop L., Groop P.-H., Handelsman Y., Insel R.A., Mathieu C. (2017). Differentiation of Diabetes by Pathophysiology, Natural History, and Prognosis. Diabetes.

[24] Maritim A.C., Sanders R.A., Watkins Iii J.B. (2003). Diabetes, oxidative stress, and antioxidants: A review. J. Biochem. Mol. Toxicol..

[25] King G.L., Loeken M.R. (2004). Hyperglycemia-induced oxidative stress in diabetic complications. Histochem. Cell Biol..

[26] Rolo A.P., Palmeira C.M. (2006). Diabetes and mitochondrial function: Role of hyperglycemia and oxidative stress. Toxicol. Appl. Pharmacol..

[27] Agada R., Usman W.A., Shehu S., Thagariki D. (2020). In vitro and in vivo inhibitory effects of *Carica papaya* seed on α-amylase and α-glucosidase enzymes. Heliyon.

[28] Raffaelli F., Nanetti L., Montecchiani G., Borroni F., Salvolini E., Faloia E., Ferretti G., Mazzanti L., Vignini A. (2015). In vitro effects of fermented papaya (*Carica papaya*, L.) on platelets obtained from patients with type 2 diabetes. Nutr. Metab. Cardiovasc. Dis..

[29] Somanah J., Aruoma O.I., Gunness T.K., Kowelssur S., Dambala V., Murad F., Googoolye K., Daus D., Indelicato J., Bourdon E. (2012). Effects of a short term supplementation of a fermented papaya preparation on biomarkers of diabetes mellitus in a randomized Mauritian population. Prev. Med..

[30] Somanah J., Bourdon E., Rondeau P., Bahorun T., Aruoma O.I. (2014). Relationship between fermented papaya preparation supplementation, erythrocyte integrity and antioxidant status in pre-diabetics. Food Chem. Toxicol..

[31] Miranda-Osorio P.H., Castell-Rodríguez A.E., Vargas-Mancilla J., Tovilla-Zárate C.A., Ble-Castillo J.L., Aguilar-Domínguez D.E., Juárez-Rojop I.E., Díaz-Zagoya J.C. (2016). Protective Action of *Carica papaya* on β-Cells in Streptozotocin-Induced Diabetic Rats. Int. J. Environ. Res. Public Health.

[32] Juárez-Rojop I.E., Díaz-Zagoya J.C., Ble-Castillo J.L., Miranda-Osorio P.H., Castell-Rodríguez A.E., Tovilla-Zárate C.A., Rodríguez-Hernández A., Aguilar-Mariscal H., Ramón-Frías T., Bermúdez-Ocaña D.Y. (2012). Hypoglycemic effect of *Carica papaya* leaves in streptozotocin-induced diabetic rats. BMC Complement. Altern. Med..

[33] Oboh G., Olabiyi A.A., Akinyemi A.J., Ademiluyi A.O. (2014). Inhibition of key enzymes linked to type 2 diabetes and sodium nitroprusside-induced lipid peroxidation in rat pancreas by water-extractable phytochemicals from unripe pawpaw fruit (*Carica papaya*). J. Basic Clin. Physiol. Pharmacol..

[34] Gella A., Durany N. (2009). Oxidative stress in Alzheimer disease. Cell Adh. Migr..

[35] Zhao Y., Zhao B. (2013). Oxidative stress and the pathogenesis of Alzheimer’s disease. Oxid. Med. Cell Longev..

[36] Huang W.-J., Zhang X., Chen W.-W. (2016). Role of oxidative stress in Alzheimer’s disease. Biomed. Rep..

[37] Zhang J., Mori A., Chen Q., Zhao B. (2006). Fermented papaya preparation attenuates beta-amyloid precursor protein: Beta-amyloid-mediated copper neurotoxicity in beta-amyloid precursor protein and beta-amyloid precursor protein Swedish mutation overexpressing SH-SY5Y cells. Neuroscience.

[38] Barbagallo M., Marotta F., Dominguez L.J. (2015). Oxidative stress in patients with Alzheimer’s disease: Effect of extracts of fermented papaya powder. Mediat. Inflamm..

[39] Highfield J. (2009). Diagnosis and classification of periodontal disease. Aust. Dent. J..

[40] Liu C., Mo L., Niu Y., Li X., Zhou X., Xu X. (2017). The Role of Reactive Oxygen Species and Autophagy in Periodontitis and Their Potential Linkage. Front. Physiol..

[41] Saliasi I., Llodra J.C., Bravo M., Tramini P., Dussart C., Viennot S., Carrouel F. (2018). Effect of a Toothpaste/Mouthwash Containing *Carica papaya* Leaf Extract on Interdental Gingival Bleeding: A Randomized Controlled Trial. Int. J. Environ. Res. Public Health.

[42] Kharaeva Z.F., Zhanimova L.R., Mustafaev M., De Luca C., Mayer W., Chung Sheun Thai J., Tiew Siok Tuan R., Korkina L.G. (2016). Effects of Standardised Fermented Papaya Gel on Clinical Symptoms, Inflammatory Cytokines, and Nitric Oxide Metabolites in Patients with Chronic Periodontitis: An Open Randomised Clinical Study. Mediat. Inflamm..

[43] Rinnerthaler M., Bischof J., Streubel M.K., Trost A., Richter K. (2015). Oxidative stress in aging human skin. Biomolecules.

[44] Masaki H. (2010). Role of antioxidants in the skin: Anti-aging effects. J. Dermatol. Sci..

[45] Jarisarapurin W., Sanrattana W., Chularojmontri L., Kunchana K., Wattanapitayakul S. (2019). Antioxidant Properties of Unripe *Carica papaya* Fruit Extract and Its Protective Effects against Endothelial Oxidative Stress. Evid. Based Complement. Altern. Med..

[46] Sanchez B., Li L., Dulong J., Aimond G., Lamartine J., Liu G., Sigaudo-Roussel D. (2019). Impact of Human Dermal Microvascular Endothelial Cells on Primary Dermal Fibroblasts in Response to Inflammatory Stress. Front. Cell Dev. Biol..

[47] Seo S.A., Ngo H.T.T., Hwang E., Park B., Yi T.-H. (2020). Protective effects of *Carica papaya* leaf against skin photodamage by blocking production of matrix metalloproteinases and collagen degradation in UVB-irradiated normal human dermal fibroblasts. S. Afr. J. Bot..

[48] Bertuccelli G., Zerbinati N., Marcellino M., Nanda Kumar N.S., He F., Tsepakolenko V., Cervi J., Lorenzetti A., Marotta F. (2016). Effect of a quality-controlled fermented nutraceutical on skin aging markers: An antioxidant-control, double-blind study. Exp. Ther. Med..

[49] Saini R., Mittal A., Rathi V. (2016). Formulation & in vitro antioxidant analysis of anti-ageing cream of *Carica papaya* fruit extract. IJOD.

[50] Magnani C., Isaac V., Corrêa M., Salgado H. (2014). Caffeic acid: A review of its potential use in medications and cosmetics. Anal. Methods.

[51] Gomes W.F., França F.R.M., Denadai M., Andrade J.K.S., da Silva Oliveira E.M., de Brito E.S., Rodrigues S., Narain N. (2018). Effect of freeze- and spray-drying on physico-chemical characteristics, phenolic compounds and antioxidant activity of papaya pulp. J. Food Sci. Technol..

[52] Nugroho A., Heryani H., Choi J.S., Park H.-J. (2017). Identification and quantification of flavonoids in Carica papaya leaf and peroxynitrite-scavenging activity. Asian Pac. J. Trop. Biomed..

[53] Spagnol C.M., Di Filippo L.D., Isaac V.L.B., Correa M.A., Salgado H.R.N. (2017). Caffeic Acid in Dermatological Formulations: In Vitro Release Profile and Skin Absorption. Comb. Chem. High Throughput Screen..

[54] Choi S.J., Lee S.N., Kim K., Joo da H., Shin S., Lee J., Lee H.K., Kim J., Kwon S.B., Kim M.J. (2016). Biological effects of rutin on skin aging. Int. J. Mol. Med..

[55] Midwood K.S., Williams L.V., Schwarzbauer J.E. (2004). Tissue repair and the dynamics of the extracellular matrix. Int. J. Biochem. Cell Biol..

[56] Gonzalez A.C., Costa T.F., Andrade Z.A., Medrado A.R. (2016). Wound healing—A literature review. Bras. Dermatol..

[57] Lephart E.D. (2016). Skin aging and oxidative stress: Equol’s anti-aging effects via biochemical and molecular mechanisms. Ageing Res. Rev..

[58] Süntar I., Akkol E.K., Nahar L., Sarker S.D. (2012). Wound healing and antioxidant properties: Do they coexist in plants?. Free Radic. Antioxid..

[59] Cano Sanchez M., Lancel S., Boulanger E., Neviere R. (2018). Targeting Oxidative Stress and Mitochondrial Dysfunction in the Treatment of Impaired Wound Healing: A Systematic Review. Antioxidants.

[60] Singh S., Young A., McNaught C.-E. (2017). The physiology of wound healing. Surg. Oxf. Int. Ed..

[61] Panzarini E., Dwikat M., Mariano S., Vergallo C., Dini L. (2014). Administration Dependent Antioxidant Effect of *Carica papaya* Seeds Water Extract. Evid. Based Complement. Alternat. Med..

[62] Mikhal’chik E.V., Ivanova A.V., Anurov M.V., Titkova S.M., Pen’kov L.Y., Kharaeva Z.F., Korkina L.G. (2004). Wound-healing effect of papaya-based preparation in experimental thermal trauma. Bull. Exp. Biol. Med..

[63] Nafiu A.B., Rahman M.T. (2015). Anti-inflammatory and antioxidant properties of unripe papaya extract in an excision wound model. Pharm. Biol..

[64] Nafiu A.B., Rahman M.T. (2015). Selenium added unripe *Carica papaya* pulp extracts enhance wound repair through TGF-β1 and VEGF-a signalling pathway. BMC Complement. Altern. Med..

[65] Collard E., Roy S. (2010). Improved function of diabetic wound-site macrophages and accelerated wound closure in response to oral supplementation of a fermented papaya preparation. Antioxid. Redox Signal..

[66] Dickerson R., Deshpande B., Gnyawali U., Lynch D., Gordillo G.M., Schuster D., Osei K., Roy S. (2012). Correction of aberrant NADPH oxidase activity in blood-derived mononuclear cells from type II diabetes mellitus patients by a naturally fermented papaya preparation. Antioxid. Redox Signal..

[67] Dickerson R., Banerjee J., Rauckhorst A., Pfeiffer D.R., Gordillo G.M., Khanna S., Osei K., Roy S. (2015). Does oral supplementation of a fermented papaya preparation correct respiratory burst function of innate immune cells in type 2 diabetes mellitus patients?. Antioxid. Redox Signal..

[68] Indran M., Mahmood A.A., Kuppusamy U.R. (2008). Protective effect of *Carica papaya* L leaf extract against alcohol induced acute gastric damage and blood oxidative stress in rats. West Indian Med. J..

[69] Nayak S.B., Pinto Pereira L., Maharaj D. (2007). Wound healing activity of *Carica papaya* L. in experimentally induced diabetic rats. Indian J. Exp. Biol..

[70] Gurung S., Skalko-Basnet N. (2009). Wound healing properties of *Carica papaya* latex: In vitro evaluation in mice burn model. J. Ethnopharmacol.

[71] Nayak B.S., Ramdeen R., Adogwa A., Ramsubhag A., Marshall J.R. (2012). Wound-healing potential of an ethanol extract of *Carica papaya* (Caricaceae) seeds. Int. Wound J..

[72] Hakim R.F., Fakhrurrazi, Dinni (2019). Effect of *Carica papaya* Extract toward Incised Wound Healing Process in Mice (Mus musculus) Clinically and Histologically. Evid. Based Complement. Alternat. Med..

[73] Ajlia S.A., Majid F.A., Suvik A., Effendy M.A., Nouri H.S. (2010). Efficacy of papain-based wound cleanser in promoting wound regeneration. Pak. J. Biol. Sci..

[74] Aravind G., Bhowmik D., S D., Harish G. (2013). Traditional and medicinal uses of *Carica papaya*. J. Med. Plants Stud..

[75] Murthy M.B., Murthy B.K., Bhave S. (2012). Comparison of safety and efficacy of papaya dressing with hydrogen peroxide solution on wound bed preparation in patients with wound gape. Indian J. Pharm..

[76] Saha S.K., Lee S.B., Won J., Choi H.Y., Kim K., Yang G.-M., Dayem A.A., Cho S.-G. (2017). Correlation between Oxidative Stress, Nutrition, and Cancer Initiation. Int. J. Mol. Sci..

[77] Sosa V., Moliné T., Somoza R., Paciucci R., Kondoh H., Lleonart M.E. (2013). Oxidative stress and cancer: An overview. Ageing Res. Rev..

[78] García-Solís P., Yahia E.M., Morales-Tlalpan V., Díaz-Muñoz M. (2009). Screening of antiproliferative effect of aqueous extracts of plant foods consumed in México on the breast cancer cell line MCF-7. Int. J. Food Sci. Nutr..

[79] Zuhrotun Nisa F., Astuti M., Murdiati A., Mubarika Haryana S. (2017). Anti-proliferation and Apoptosis Induction of Aqueous Leaf Extract of *Carica papaya* L. on Human Breast Cancer Cells MCF-7. Pak. J. Biol. Sci..

[80] Pathak N., Khan S., Bhargava A., Raghuram G.V., Jain D., Panwar H., Samarth R.M., Jain S.K., Maudar K.K., Mishra D.K. (2014). Cancer chemopreventive effects of the flavonoid-rich fraction isolated from papaya seeds. Nutr. Cancer.

[81] Somanah J., Ramsaha S., Verma S., Kumar A., Sharma P., Singh R.K., Aruoma O.I., Bourdon E., Bahorun T. (2016). Fermented papaya preparation modulates the progression of N-methyl-N-nitrosourea induced hepatocellular carcinoma in Balb/c mice. Life Sci..

[82] Waly M.I., Al-Rawahi A.S., Al Riyami M., Al-Kindi M.A., Al-Issaei H.K., Farooq S.A., Al-Alawi A., Rahman M.S. (2014). Amelioration of azoxymethane induced-carcinogenesis by reducing oxidative stress in rat colon by natural extracts. BMC Complement. Altern. Med..

[83] Murakami S., Eikawa S., Kaya S., Imao M., Aji T. (2016). AntiTumor and Immunoregulatory Effects of Fermented Papaya Preparation (FPP: SAIDOPS501). Asian Pac. J. Cancer Prev..

[84] Bussmann R.W., Malca G., Glenn A., Sharon D., Nilsen B., Parris B., Dubose D., Ruiz D., Saleda J., Martinez M. (2011). Toxicity of medicinal plants used in traditional medicine in Northern Peru. J. Ethnopharmacol..

[85] Afzan A., Abdullah N.R., Halim S.Z., Rashid B.A., Semail R.H.R., Abdullah N., Jantan I., Muhammad H., Ismail Z. (2012). Repeated dose 28-days oral toxicity study of *Carica papaya* L. leaf extract in Sprague Dawley rats. Molecules.

[86] Halim S.Z., Abdullah N., Afzan A., Abd Rashid B., Jantan I., Ismail Z. (2011). Acute toxicity study of *Carica papaya* leaf extract in Sprague Dawley rats. J. Med. Plants Res..

[87] Tarkang P., Agbor G., Armelle T., Tchokouaha L.R., David K., Ngadena Y. (2012). Acute and Chronic Toxicity Studies of the aqueous and ethanol leaf extracts of *Carica papaya* Linn in Wistar rats. J. Nat. Prod. Plant Resour..

